# The Burden of Informal Caregivers of People with Dementia: An Integrative Review

**DOI:** 10.1002/alz70858_107599

**Published:** 2025-12-26

**Authors:** Isabela Alicia Fink, Laura Carolina Nardi Motta, Maria Fernanda Peruci Felippe, Matias Pinheiro de Macêdo Neto, Ana Carolina Gomes Petry, Gabriela Bezerra Sorato, Jessica Vargas Lopes, Daniel Pasini Gonçalves, Vitório Serafim, Morghana Machado da Rosa

**Affiliations:** ^1^ Fundação Universidade Federal de Ciências da Saúde de Porto Alegre, Porto Alegre, Rio Grande do Sul, Brazil; ^2^ Universidade Luterana do Brasil, Canoas, Rio Grande do Sul, Brazil

## Abstract

**Background:**

Dementia is a progressive neurodegenerative syndrome affecting millions, increasing the demand for informal caregiving. Informal caregivers, primarily family members, play a crucial role in supporting individuals with dementia, often facing substantial emotional, physical, and social burdens. This study analyzes the impact of caregiver burden on health and well‐being, considering individual, contextual, and cultural influences.

**Method:**

An integrative literature review was conducted using a systematic search in the PubMed database. Inclusion criteria covered studies published between 2014 and 2024 focusing on informal caregivers of people with dementia. A total of 29 studies were selected and analyzed to synthesize findings related to mental and physical health, quality of life, caregiver characteristics, sociocultural context, anticipatory grief, and positive aspects of caregiving.

**Result:**

Caregiver burden is a multidimensional phenomenon with significant effects on health and well‐being. Mental health impacts were predominant, with high rates of depression (reported in up to 60% of caregivers in some studies), anxiety, stress, and emotional exhaustion. Burnout and anticipatory grief were also frequently described, especially in caregivers witnessing the progressive cognitive and functional decline of loved ones. Physical health consequences included fatigue, sleep disturbances, chronic pain, and a decline in overall functional status. Some studies indicated an increased risk of mortality among caregivers due to prolonged stress and neglect of self‐care. Quality of life was negatively affected in multiple dimensions, including social relationships, financial stability, and well‐being. Sociocultural factors, such as gender roles, familial expectations, and economic conditions, influenced the caregiving experience, with women disproportionately affected. The COVID‐19 pandemic exacerbated caregiver burden, increasing social isolation, reducing healthcare access, and heightening psychological distress. Despite these challenges, some caregivers reported personal growth, stronger family bonds, and a sense of purpose.

**Conclusion:**

Informal caregiving for individuals with dementia imposes a substantial burden, affecting mental and physical health and overall quality of life. The findings underscore the urgent need for targeted interventions, including mental health support, respite care, and policies addressing financial and social challenges. Future research should explore long‐term caregiver outcomes and strategies to enhance resilience.

